# Supplementary material for: The CD2 isoform of protocadherin-15 is an essential component of the tip-link complex in mature auditory hair cells
Source: EMBO Mol Med. 2014 Jun 17;6(7):984–92. doi: 10.15252/emmm.201403976 (PMC4119359; doi:10.15252/emmm.201403976)
Supplement: Supplementary file 1 — Supplementary Figure S1 [file emmm0006-0984-SD1.pdf]

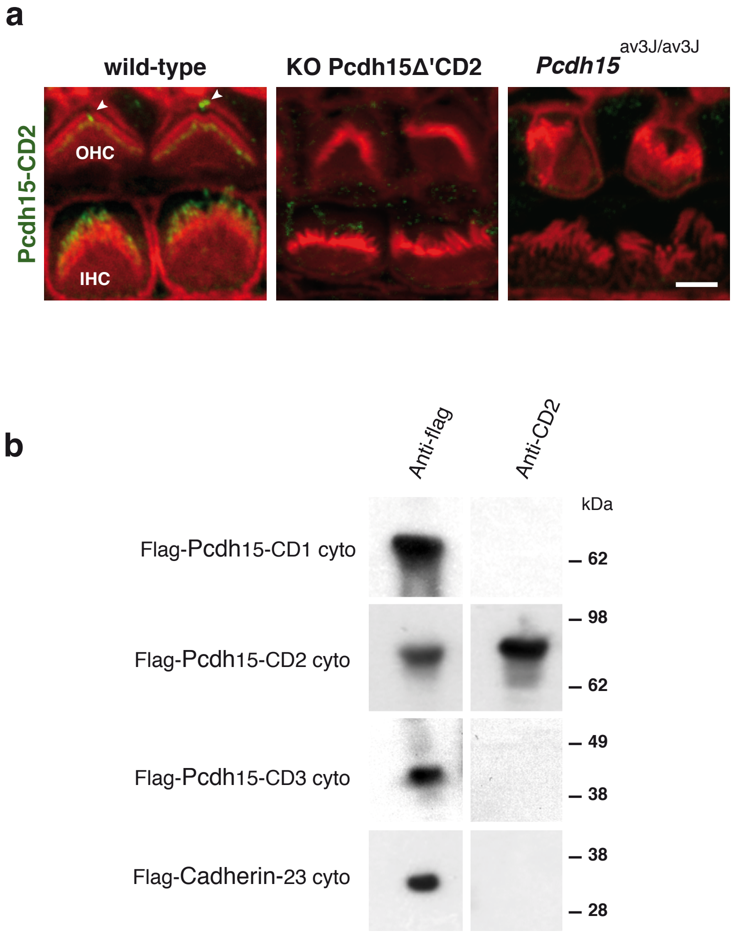

Supplementary Figure S1: Specificity of the anti-Pcdh15-CD2 antibody

(a) Confocal images of whole-mount preparations of cochlear sensory epithelia from P5 wild-type, KO Pcdh15 Δ'CD2 and *Pcdh15* $^{av3J/av3J}$ mice (see Methods and Supporting Information, Fig 3) stained for Pcdh15-CD2 (green) and actin (red). Pcdh15-CD2 labelling is evident in the stereocilia and kinocilia (arrowheads) of the wild-type mouse but not of KO Pcdh15Δ'CD2 or *Pcdh15* $^{av3J/av3J}$ mice.

Scale bar: 2 μm

(b) Western blot showing the specificity of the anti-Pcdh15-CD2 antibody (Flag-Pcdh15-CD1 cyto, Flag-Pcdh15-CD2, Flag-Pcdh15-CD3 cyto and Flag-Cadherin23-cyto denote the flag-tagged cytoplasmic domains of Pcdh15-CD1, Pcdh15-CD2, Pcdh15-CD3, and cadherin-23, respectively).
