# Supplementary material for: The CD2 isoform of protocadherin-15 is an essential component of the tip-link complex in mature auditory hair cells
Source: EMBO Mol Med. 2014 Jun 17;6(7):984–92. doi: 10.15252/emmm.201403976 (PMC4119359; doi:10.15252/emmm.201403976)
Supplement: Supplementary file 2 — Supplementary Figure S2 [file emmm0006-0984-SD2.pdf]

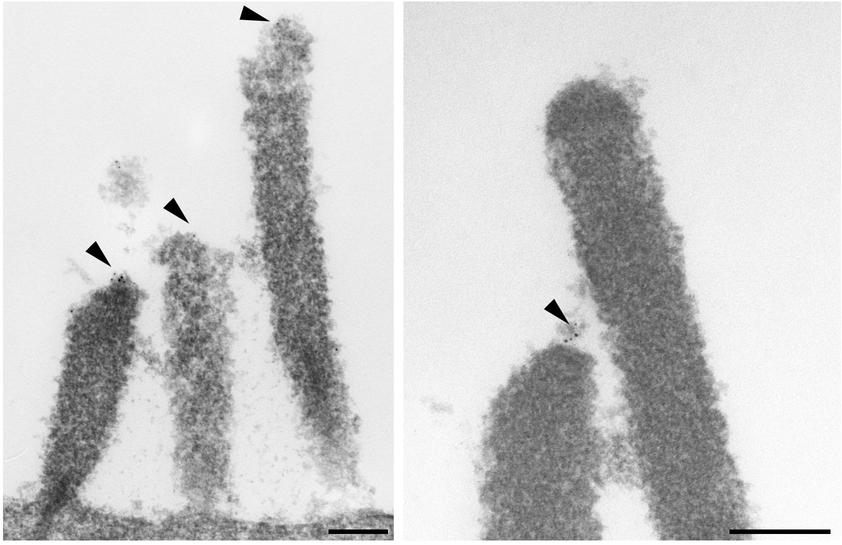

Supplementary Figure S2: Immunogold labelling of Pcdh15-CD2 in a murine OHC

Transmission electron micrograph of a Pcdh15-CD2 immunoreactive OHC hair bundle at P11 (left panel), and tip-link profile from an OHC at P15 (right panel). Arrowheads indicate gold particles that are located at the stereocilia tips.

Scale bars: 200 nm
