# Supplementary material for: The CD2 isoform of protocadherin-15 is an essential component of the tip-link complex in mature auditory hair cells
Source: EMBO Mol Med. 2014 Jun 17;6(7):984–92. doi: 10.15252/emmm.201403976 (PMC4119359; doi:10.15252/emmm.201403976)
Supplement: Supplementary file 3 — Supplementary Figure S3 [file emmm0006-0984-SD3.pdf]

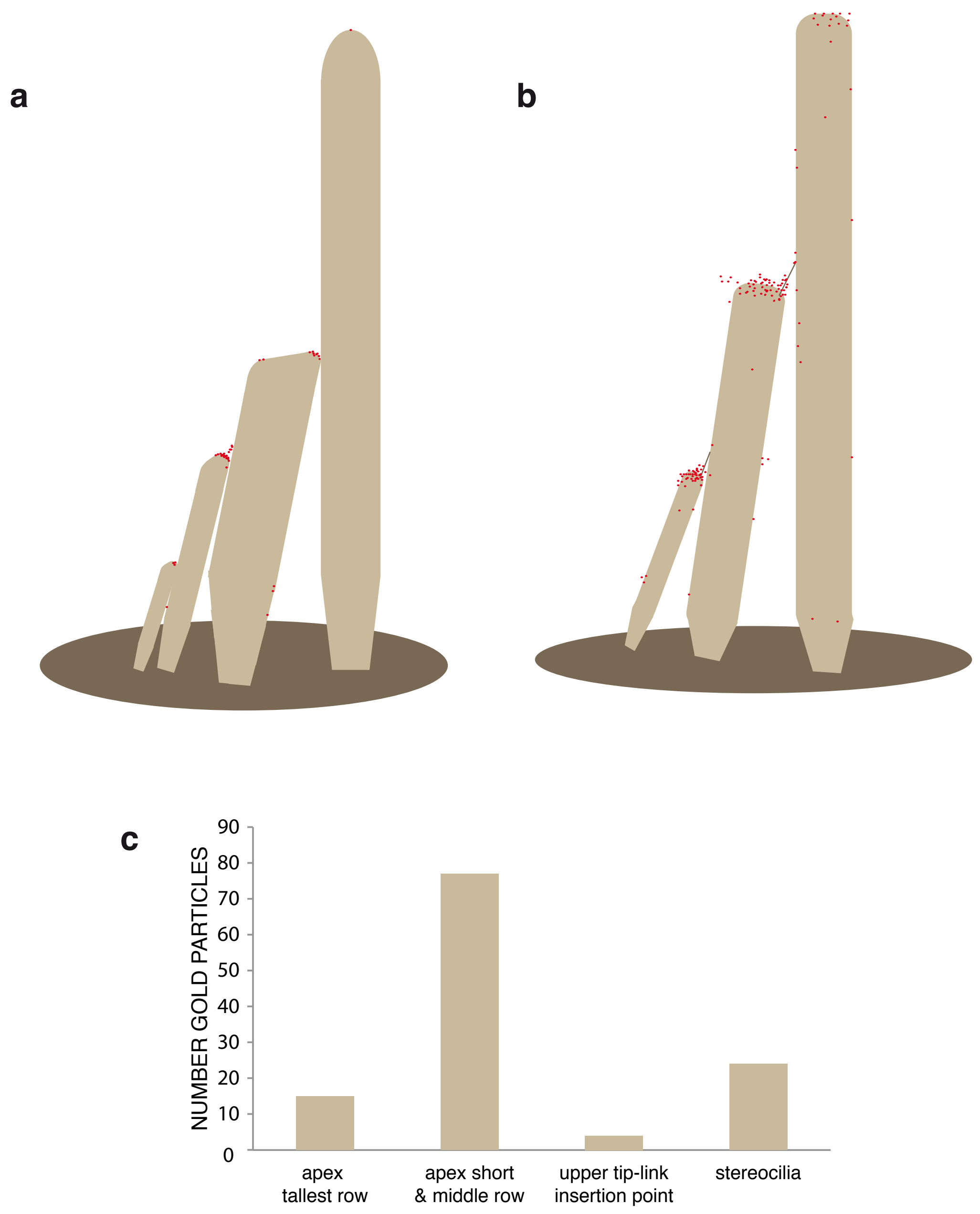

Supplementary Figure S3: Distribution of gold particles in Pcdh15-CD2 immunoreactive hair bundles

(a) The distribution of gold particles in 9 sections of mature inner hair cells (n=39).
(b) The distribution of gold particles in 16 sections of mature outer hair cells (n= 120).
(c) Quantification of gold particles in outer hair cells is shown with histograms .
