# Supplementary material for: The CD2 isoform of protocadherin-15 is an essential component of the tip-link complex in mature auditory hair cells
Source: EMBO Mol Med. 2014 Jun 17;6(7):984–92. doi: 10.15252/emmm.201403976 (PMC4119359; doi:10.15252/emmm.201403976)
Supplement: Supplementary file 4 — Supplementary Figure S4 [file emmm0006-0984-SD4.pdf]

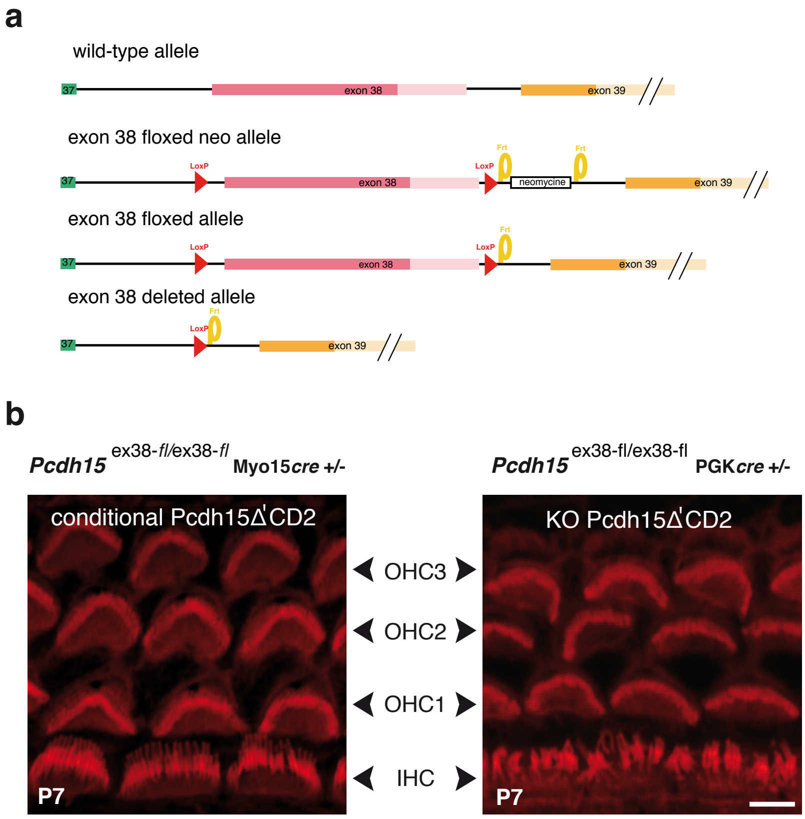

Supplementary Figure S4: Engineering conditional knockout mice lacking Pcdh15 exon 38

(a) Schematic of the exon38-recombinant alleles of Pcdh15 (see Methods)

(b) Confocal images of whole mount preparations of cochlear sensory epithelia from conditional Pcdh15 Δ'CD2 and KO Pcdh15Δ'CD2 P7 mice obtained by crossing *Pcdh15* $^{ex38\text{-}fl/ex38\text{-}fl}$ mice with *Myo15-cre* and *PGK-cre* mice, respectively (Caberlotto et al, 2011; Lallemand et al, 1998). Actin staining (red). The hair bundles of KO Pcdh15Δ'CD2 OHCs, but not of conditional Pcdh15Δ'CD2 OHCs, show planar polarity defects and abnormal shape of the OHC hair bundles similar to those described previously in knockout mice lacking Pcdh15-CD2 (PCDH15-ΔCD2) (Webb et al, 2011).

Scale bar: 2 μm
