# Supplementary material for: The CD2 isoform of protocadherin-15 is an essential component of the tip-link complex in mature auditory hair cells
Source: EMBO Mol Med. 2014 Jun 17;6(7):984–92. doi: 10.15252/emmm.201403976 (PMC4119359; doi:10.15252/emmm.201403976)
Supplement: Supplementary file 5 — Supplementary Figure S5 [file emmm0006-0984-SD5.pdf]

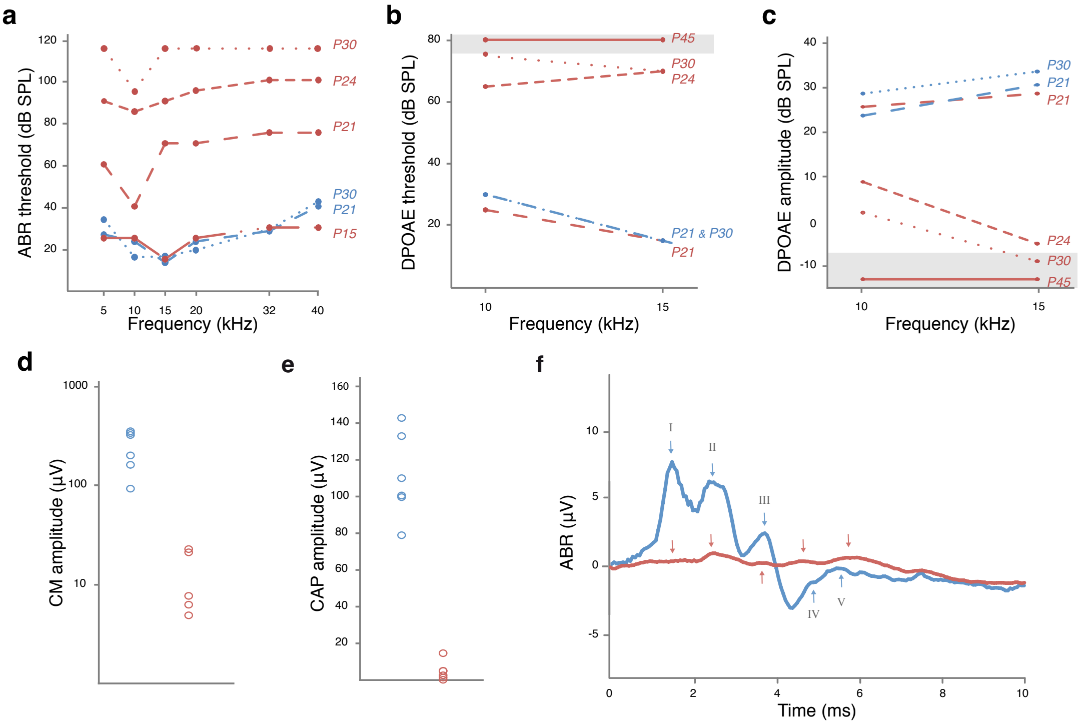

Supplementary Figure S5: Progression of auditory dysfunction in conditional Pcdh15Δ'CD2 mice

(a) Auditory brainstem response (ABR) thresholds across the frequency spectrum (from 5 to 40 kHz) at different stages from P15 to P30. Blue curves: a *Pcdh15* $^{\text{ex38-fl/ex38-fl}}$ (control) mouse; red curves: a representative conditional Pcdh15-Δ'CD2 mouse. At P15, the conditional Pcdh15Δ'CD2 mouse cannot be distinguished from the control mouse. From P21 onwards, ABR thresholds increase progressively in the conditional Pcdh15-Δ'CD2 mouse relative to the control, indicating the onset of Pcdh15-CD2 loss.

(b) Distortion product otoacoustic emission (DPOAE) thresholds measured at 10 kHz and 15 kHz. The blue and red plots correspond to a *Pcdh15* $^{\text{ex38-fl/ex38-fl}}$ (control) mouse and a representative conditional Pcdh15Δ'CD2 mouse, respectively, repeatedly tested at various stages from P21 to P45. On P21, the conditional Pcdh15 Δ'CD2 mouse cannot be distinguished from the control mouse. From P24 onwards, DPOAE thresholds increase progressively, indicating Pcdh15-CD2 loss in OHCs. DPOAEs with detection thresholds > 75 dB SPL do not differ significantly from instrumental distortion (grey area).

(c) DPOAE maximal amplitudes measured at 10 kHz and 15 kHz in response to 70 dB SPL stimuli. The blue and red plots correspond to a *Pcdh15* $^{\text{ex38-fl/ex38-fl}}$ (control) mouse and a representative conditional Pcdh15-Δ'CD2 mouse, respectively, repeatedly tested at various stages from P21 to P45. On P21 the conditional Pcdh15Δ'CD2 mouse cannot be distinguished from the control mouse. Later, DPOAE amplitudes decrease progressively, indicating Pcdh15-CD2 loss in OHCs. Signals with amplitudes < -7 dB SPL do not significantly differ from background noise (grey area).

(d) Amplitude of the cochlear microphonic (CM) response to a 10 kHz, 105 dB tone burst in control *Pcdh15* $^{\text{ex38-fl/ex38-fl}}$ (blue) and conditional Pcdh15Δ'CD2 (red) mice at P30. The amplitude is substantially lower in conditional Pcdh15Δ'CD2 mice (9.2 ± 2.6 µV, n=5) than *Pcdh15* $^{\text{ex38-fl/ex38-fl}}$ control mice (244.2 ± 42.9 µV, n=6; Student's t-test, p=0.003).

(e) Amplitude of the compound action potential (CAP) response to a 10 kHz, 105 dB tone burst in control *Pcdh15* $^{\text{ex38-fl/ex38-fl}}$ (blue) and conditional Pcdh15Δ'CD2 (red) mice at P30. The amplitude is substantially lower in conditional Pcdh15Δ'CD2 mice (4.6 ± 2.5 µV, n=5) than *Pcdh15* $^{\text{ex38-fl/ex38-fl}}$ control mice (111.0 ± 10.5 µV, n=6; Student's t-test, p<0.001).

(f) Auditory brainstem response (ABR) trace in response to a 10 kHz, 105 dB tone in a *Pcdh15* $^{\text{ex38-fl/ex38-fl}}$ (control) mouse (blue) and a representative conditional Pcdh15-Δ'CD2 mouse (red) at P30.
The amplitude of the first wave (I) was substantially lower in conditional Pcdh15Δ'CD2 mice (0.16 ± 0.06 µV, n=5) than control *Pcdh15* $^{\text{ex38-fl/ex38-fl}}$mice (6.5 ± 0.5 µV, n=5; Student's t-test, p<0.001), resulting in the subsequent waves (II to V) having small amplitudes.
