# Supplementary material for: The CD2 isoform of protocadherin-15 is an essential component of the tip-link complex in mature auditory hair cells
Source: EMBO Mol Med. 2014 Jun 17;6(7):984–92. doi: 10.15252/emmm.201403976 (PMC4119359; doi:10.15252/emmm.201403976)
Supplement: Supplementary file 6 — Supplementary Figure S6 [file emmm0006-0984-SD6.pdf]

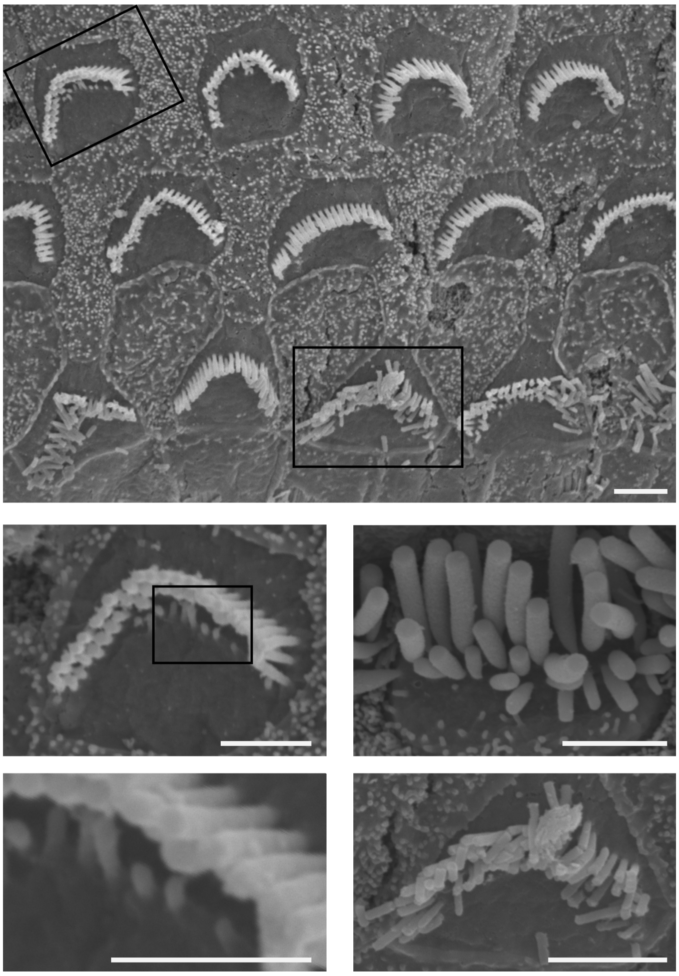

Supplementary Figure S6: Morphological analysis of hair bundles in a KO Pcdh15Δ'CD2 mouse

Scanning electron micrograph showing the morphology of hair bundles in a KO Pcdh15Δ'CD2 mouse on P30. Some OHC hair bundles show show planar polarity defects. In OHCs no tip-links are visible, and short and middle row stereocilia have regressed. In IHCs the middle row stereocilia have lost their distal prolate shape indicating tip-link disruption. The short row of stereocilia has completely regressed.

Scale bar: 1 μm
